# Walking Stability and Kinematic Variability Following Motor Fatigue Induced by Incline Treadmill Walking †

**DOI:** 10.3390/s25051489

**Published:** 2025-02-28

**Authors:** Pei-Chun Kao, Colin Lomasney

**Affiliations:** 1 Department of Physical Therapy and Kinesiology, University of Massachusetts Lowell, Lowell, MA 01854, USA; clomasney@bidmc.harvard.edu; 2 New England Robotics Validation and Experimentation (NERVE) Center, University of Massachusetts Lowell, Lowell, MA 01852, USA

**Keywords:** motor fatigue, walking, whole-body fatigue, localized fatigue, gait stability, gait variability

## Abstract

Detecting motor fatigue during rigorous activities is essential for preventing injuries, falls, and over-exertion. While research has focused on developing fatigue indices using motion capture or wearable sensors, the method of inducing fatigue can impact movement patterns differently. This study compared the effects of whole-body motor fatigue induced by incline treadmill walking with localized fatigue induced by leg presses and isolated ankle movements, as investigated in our prior study. Twenty healthy young participants walked at 1.25 m/s for 5 min before (PRE) and after (POST) motor fatigue. We computed POST-to-PRE ratios for walking stability and variability measures, including dynamic margins of stability (MOS), step spatiotemporal measures, kinematic variability, and local dynamic stability based on short-term local divergence exponents (LDEs) of trunk movement. Localized fatigue increased mean step width (*p* = 0.002), mean mediolateral MOS (*p* = 0.015), knee joint angle variability (*p* < 0.001), and the mean peak mediolateral center of mass velocity (*p* < 0.001) more than whole-body fatigue. Whole-body fatigue reduced short-term LDE values of anterior–posterior trunk motion (*p* = 0.021), indicating greater improvement in local dynamic stability. The findings indicate that localized fatigue has a greater impact on gait variability and stability than whole-body fatigue. The methods of inducing motor fatigue led to different changes in gait.

## 1. Introduction

Detecting and monitoring motor fatigue in individuals engaged in rigorous physical activities (e.g., prolonged walking, running, or tasks requiring repetitive lifting or squatting) may help mitigate risks such as musculoskeletal injuries, falls, and over-exertion. This enables timely intervention and the implementation of preventive measures. Significant research efforts have been dedicated to developing motor fatigue indices or models using movement data collected from optical motion capture or wearable sensors during physical activities [1,2,3,4]. However, the nature of motor fatigue, whether whole-body or localized, can lead to varying changes in movement patterns during these activities [5]. “Whole-body” motor fatigue typically results from exercises engaging the entire body, such as running or prolonged walking [6,7,8,9]. Conversely, “localized” motor fatigue often follows activities targeting specific muscle groups under load, such as isokinetic knee extension, leg presses, sit-to-stands, or heel raises [10,11,12,13,14]. Therefore, obtaining a comprehensive understanding of how motor fatigue impacts movement patterns is essential for enhancing prediction accuracy and injury prevention strategies.

While the effects of motor fatigue on walking kinematics have been studied extensively, research employing various methods to induce fatigue has produced mixed results [5]. Following localized fatiguing exercises, healthy participants tend to walk with reduced speed and/or step length [11,14], along with increased step width and variability in stride parameters and trunk movement profile [11,12]. Although fewer studies have examined whole-body fatigue effects on walking kinematics, similar changes in stride variability have been observed [6,15]. However, there is less consensus on changes in walking stability following motor fatigue. Some studies report that healthy participants maintain a similar level of walking stability following localized fatigue [11,12]. Suzuki et al (2014) [16] demonstrated improved walking stability following whole-body fatigue when participants maintained the same treadmill speed. Yoshino et al (2004) examined self-paced overground walking and found that, following whole-body fatigue, some participants reduced walking speed to improve walking stability, while others maintained walking speed but reduced walking stability [15]. Hamacher et al (2018) reported similar walking stability after whole-body fatigue [9].

It remains unclear how whole-body versus localized motor fatigue impacts walking performance differently. Previous studies employed various outcome measures to evaluate the effects of motor fatigue, complicating the comparisons [5,17]. In addition, some studies used self-paced overground walking to examine walking performance, which did not control walking speeds across pre- and post-fatigue conditions [9,14,15]. Notably, walking speed alone can influence walking stability and variability, even in the absence of motor fatigue [18,19,20]. Building upon our previous study, which investigated localized motor fatigue induced by leg presses and isolated ankle plantar flexion and dorsiflexion movements [12], the current study focused on whole-body motor fatigue induced by incline treadmill walking [21]. Specifically, this study examined the effects of whole-body fatigue on gait stability and variability. Furthermore, we compared the alterations in walking performance following whole-body fatigue with those observed in localized fatigue by integrating findings from both studies. We hypothesized that whole-body motor fatigue would have a greater impact on gait variability and stability compared to localized motor fatigue. This is because whole-body activities are likely to disrupt a broader range of sensory systems, including visual, vestibular, and plantar cutaneous mechano-receptors, compared to localized exercises [22,23,24].

## 2. Methods

### 2.1. Participants

Twenty healthy young participants (nine females: age, 24.0 ± 7.0 years; body height, 1.61 ± 0.07 m; body mass, 62.3 ± 8.6 kg. Eleven males: age, 22.8 ± 2.8 years; body height, 1.80 ± 0.09 m; body mass, 86.9 ± 12.1 kg; mean ± STD) gave written informed consent to participate in the study, which was approved by the Institutional Review Board of the University (#20-057). Participants were excluded if they had any medical condition, history of major leg injuries, or pain in the legs or spine that limits locomotion or the capacity to perform exercise.

### 2.2. Experimental Procedures

Participants performed 3 maximal vertical squat jumps (non-countermovement) with one arm crossed over the chest and the other arm tapping the Vertec vertical jump tester (JumpUSA, Sunnyvale, CA, USA). The highest jump height was recorded. Before and after the fatiguing protocol, participants walked on a treadmill at 1.25 m/s and 0° incline for five minutes. The procedures for inducing whole-body fatigue using the incline treadmill walking protocol have been detailed previously [25]. Briefly, during the protocol, the treadmill inclination increased by 2.5° every five minutes until participants reached a Borg rating of perceived exertion (RPE) > 17/20 and ~85% of their maximum age-predicted heart rate or they requested to stop due to fatigue (i.e., voluntary exhaustion). At this point, participants performed another 2–3 squat jumps. If jump height decreased by 20%, the fatiguing protocol ended. Otherwise, participants resumed walking from the last incline setting achieved and continued until the pausing criteria were met again. Once all criteria were met, participants walked for one minute at the last incline setting before proceeding to the post-fatigued walking trial at 0° incline.

The procedures to induce localized motor fatigue were detailed in our previous study, using leg presses and isolated ankle movements [12]. Briefly, participants performed leg presses at 80% of their one-repetition maximum (1-RM) at 50 beats per minute (bpm) for 2 min, or until voluntary exhaustion. After a one-minute rest, participants performed two consecutive leg presses at 85% of their 1-RM or 80% of 1-RM plus 20 lbs. If participants could only perform one leg press or none against this load, the leg press exercise ended. Otherwise, additional bout(s) of leg presses at 80% of 1-RM would be conducted until this criterion was met. Afterward, participants performed calf raises at 90 bpm with a preferred dumbbell weight and seated toe raises against a Theraband until voluntary exhaustion. Participants walked on the treadmill at 1.25 m/s for three minutes both before and after the exercise session.

### 2.3. Data Collection and Analysis

We recorded three-dimensional kinematics using an 8-camera video system (100 Hz, Motion Analysis Corporation, Santa Rosa, CA, USA) with reflective markers attached to the lower body, trunk, and the back of the neck (C7 vertebra) as participants walked on the treadmill at 0° inclination before (PRE) and after (POST) the motor fatiguing protocol. We used Visual 3D software (C-Motion Inc., Germantown, MD, USA) to perform initial data processing, such as filtering the marker data, gait event detection, gait cycle normalization, computation of joint angle at the ankle, knee, and hip, as well as deriving the center of mass position and velocity.

We quantified walking stability by computing the short-term local divergence exponents (LDEs) of trunk motion (i.e., local dynamic stability) using the reconstructed state spaces of C7 vertebral marker data [26]. Short-term LDEs measure the sensitivity of the human locomotor system to naturally occurring perturbations during walking. For LDE computation, we extracted 240 continuous strides for each trial and resampled the data so that each stride would have approximately 100 data points (i.e., 240 strides for 24,000 total points). Delay embedded state spaces with an embedding dimension of 5 were reconstructed independently from the mediolateral (M-L) and anterior–posterior (A-P) velocities of the non-filtered C7 vertebral marker data and their time delayed copies. We examined trunk motions because maintaining dynamic stability of the upper body is critical in human locomotion [27]. In addition, trunk motion dynamics have been suggested to provide a more sensitive marker for assessing the changes in gait stability compared to other body segments [28] and C7 marker data have been used in previous studies to represent the trunk motion [26,29,30]. Fixed time delays of 30 and 24 data samples (i.e., 30% and 24% of the stride cycle) were used for the M-L and A-P directions, respectively. Procedures to compute short-term LDEs were well established and stated in previous studies [12,19,26,29,30,31]. LDEs quantified how quickly neighboring movement trajectories in a state space diverge over time. A positive, larger value of short-term LDE indicates greater instability to local perturbations.

We also computed the mean and standard deviation of the dynamic margins of stability (MOS) [32] to quantify instantaneous walking stability. We derived the anterior–posterior and mediolateral MOS (MOS_AP_ and MOS_ML_) by calculating the distance between the extrapolated position of the center of mass (COM) [32] and the front and lateral toe markers of the leading foot, respectively. To account for the relative motion of the treadmill belt during walking, we added belt velocity to the anterior–posterior COM velocity to compute the extrapolated COM position [33,34] and thus, we recalculated MOS_AP_ for the localized fatigue data from our previous study [12]. MOS_AP_ and MOS_ML_ were computed at heel strikes of each foot for each trial. Procedures to compute MOS were well established and stated in previous research [33,34].

We computed the mean and standard deviation of the spatiotemporal step measures (i.e., step width, step length, and stride time) and kinematic variability for each walking trial. For kinematic variability, the mean standard deviation (meanSD) of the COM position, COM velocity, and lower-extremity joint kinematics was calculated across strides at each normalized time point (0–100%) of the gait cycle and then averaged over the whole gait cycle to produce a single measure of the mean variability for each trial [18]. Additionally, we computed the meanSD of the C7 marker positions and velocities to quantify the overall variability of participants’ displacements (i.e., drift) on the treadmill and stride-to-stride trunk movement variability, consistent with our prior methodology. Subsequently, these values were averaged over the gait cycle to derive a single measure of the mean variability for each trial. Furthermore, we computed the mean peak COM velocity during the gait cycle for each trial, representing the magnitude of trunk sways.

To compare with the localized motor fatigue data published in our prior study [12], we also computed POST to PRE ratios for all parameters.

### 2.4. Statistical Analysis

We conducted Shapiro–Wilk tests to assess the normality of all outcome measures. Most outcome measures were normally distributed, except for the POST/PRE ratios of mean MOS_AP_ and STD MOS_ML_. We used paired *t*-tests to test for differences in outcome variables before and after the whole-body motor fatiguing protocol (i.e., PRE and POST, respectively). We used independent *t*-tests to test for differences in the POST/PRE ratios of gait parameters that were normally distributed between the whole-body and localized fatigue protocols. For the non-normally distributed POST/PRE ratios of mean MOS_AP_ and STD MOS_ML_, we performed Mann–Whitney U tests. We set the significance level at 0.05. All statistical analyses were performed in SPSS version 28 (IBM, New York, NY, USA).

## 3. Results

### 3.1. Performance Following the Whole-Body Motor Fatiguing Protocol

The accumulated treadmill walking time to induce whole-body motor fatigue was 37.4 ± 8.6 min, excluding the five-minute post-fatigue walking trial. The inclination angle achieved was 5° for one participant, 7.5° for nine participants, and 10° for ten participants.

#### 3.1.1. Walking Stability and Step Measures

For local dynamic stability (Table 1), participants showed significantly smaller short-term LDE values for trunk motion in both directions during POST compared to PRE (A-P: *p* = 0.001, M-L: *p* = 0.039), indicating increased stability post-fatigue. For dynamic margins of stability, participants walked with a significantly greater mean MOS_AP_ (*p* < 0.001) but exhibited similar variability in MOS_AP_ (*p* = 0.155) during POST compared to PRE. Conversely, participants walked with a similar mean MOS_ML_ (*p* = 0.448) but had significantly greater MOS_ML_ variability (*p* = 0.016) during POST.

For mean step measures, compared to PRE, participants exhibited a trend toward increased mean step length (*p* = 0.058) but reduced mean step width (*p* = 0.051), with similar stride time (*p* = 0.284) during POST. Regarding step variability, participants showed a trend toward reduced step length variability (*p* = 0.070) and stride time variability (*p* = 0.064), but they exhibited significantly increased step width variability (*p* = 0.005) during POST.

#### 3.1.2. Joint Angle Profile and Kinematic Variability

**Figure 1:**
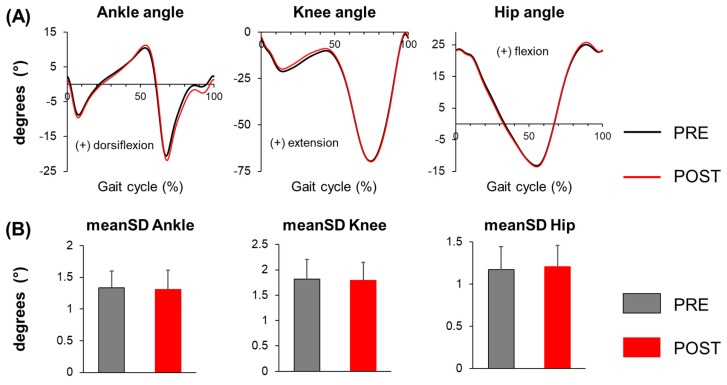
(**A**) Joint angle profiles of the ankle, knee, and hip, and (**B**) mean standard deviation (meanSD) of the ankle, knee, and hip joint angles before (PRE) and after (POST) the whole-body motor fatiguing protocol. Error bars represent ± 1 STD.

In the average joint angle profile (Figure 1), participants exhibited an increased peak ankle dorsiflexion angle at mid-stance (PRE: 10.5° ± 3.0°, POST: 11.3° ± 3.1°, *p* = 0.014), greater peak ankle plantar flexion angle during push-off (PRE: −20.9° ± 4.1°, POST: −22.2° ± 4.4°, *p* = 0.028), and increased ankle plantar flexion at mid-swing (PRE: −0.3° ± 4.2°, POST: −1.8° ± 4.4°, *p* < 0.001) during POST compared to PRE. However, knee and hip joint angle profiles were similar during POST, except for a trend towards reduced peak knee flexion angle in early stance (PRE: −21.3° ± 4.2°, POST: −20.2° ± 4.7°, *p* = 0.072). Joint angle variability for all three joints remained similar during POST compared to PRE (Table 1).

Compared to PRE, participants showed significantly increased COM position variability in both the A-P (*p* = 0.043) and M-L (*p* < 0.001) directions during POST, indicating a greater overall variability of their displacements on the treadmill (i.e., drift) after fatigue (Table 1). Additionally, participants exhibited similar COM velocity variability in the A-P direction (*p* = 0.295), but significantly greater variability in the M-L direction (*p* < 0.001) during POST, suggesting increased stride-to-stride variability in M-L trunk movement post-fatigue. For mean peak COM velocity, on the contrary, participants exhibited significantly greater peak COM velocity in the A-P direction (*p* = 0.006) but similar peak COM velocity in the M-L direction (*p* = 0.134) during walking at POST, indicating increased trunk movement primarily in the A-P direction after fatigue.

### 3.2. Comparisons Between Whole-Body Versus Localized Motor Fatigue

#### 3.2.1. POST/PRE Ratios of Walking Stability and Step Measures

**Figure 2:**
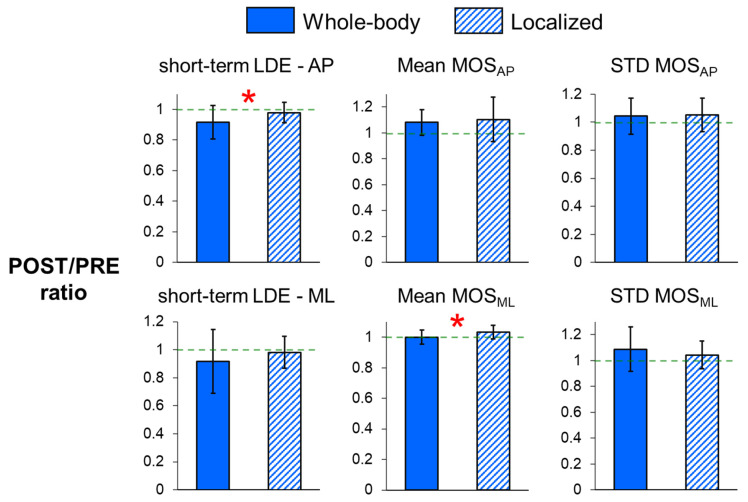
POST/PRE ratios of short-term local divergence exponents (LDEs) in the anteroposterior (A-P) and mediolateral (M-L) directions, and POST/PRE ratios of mean and variability in MOS_AP_ and MOS_ML_ for whole-body and localized motor fatigue protocols. The POST/PRE ratios represent the ratio of values for these measures in the post-fatigue (POST) condition relative to the pre-fatigue (PRE) condition. Error bars represent ± 1 STD. * indicates a significant difference between the whole-body and localized fatigue conditions (*p* < 0.05).

POST/PRE ratios of short-term LDE measures were all smaller than one for both whole-body and localized fatigue (Table 2, Figure 2). In addition, whole-body fatigue exhibited a significantly smaller POST/PRE ratio for the short-term LDE value of A-P trunk motion (*p* = 0.021) compared to localized fatigue. These results indicate that A-P local stability improved to a greater extent following whole-body fatigue than localized fatigue.

**Figure 3:**
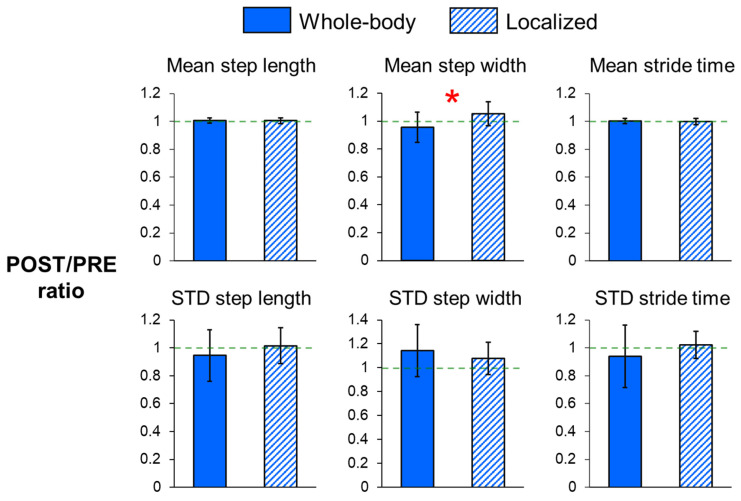
POST/PRE ratios of mean and variability in step length, step width, and stride time for whole-body and localized motor fatigue protocols. The POST/PRE ratios represent the ratio of values for these measures in the post-fatigue (POST) condition relative to the pre-fatigue (PRE) condition. Error bars represent ± 1 STD. * indicates significant difference between the whole-body and localized fatigue conditions (*p* < 0.05).

For the dynamic margins of stability (Figure 2) and step measures (Figure 3), the POST/PRE ratios of mean MOS_ML_ (*p* = 0.015) and mean step width (*p* = 0.002) were significantly higher for localized fatigue compared to whole-body fatigue, indicating a greater increase in mean MOS_ML_ and mean step width following localized fatigue. Additionally, there was a trend toward smaller POST/PRE ratios in step length variability (*p* = 0.094) and stride time variability (*p* = 0.078) for whole-body fatigue compared to localized fatigue.

#### 3.2.2. POST/PRE Ratios of Kinematic Variability

**Figure 4:**
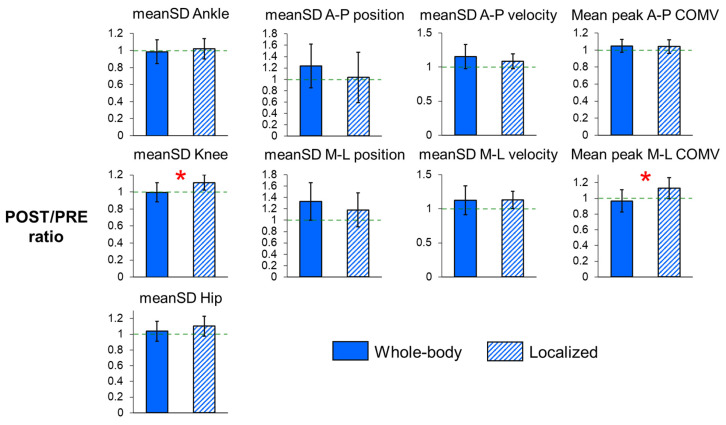
POST/PRE ratios of meanSD of the ankle, knee, and hip angles, POST/PRE ratios of meanSD of C7 vertebral marker position and velocity, and POST/PRE ratios of mean peak center of mass (COM) velocity in the A-P and M-L directions for whole-body and localized motor fatigue protocols. The POST/PRE ratios represent the ratio of values for these measures in the post-fatigue (POST) condition relative to the pre-fatigue (PRE) condition. Error bars represent ± 1 STD. * indicates significant difference between the whole-body and localized fatigue conditions (*p* < 0.05).

For joint angle variability (Table 2, Figure 4), the POST/PRE ratio of knee angle variability (*p* < 0.001) was significantly greater for localized fatigue compared to whole-body fatigue. Additionally, there was a trend toward a higher POST/PRE ratio in hip angle variability (*p* = 0.064) after localized fatigue. Conversely, whole-body fatigue showed trends toward increased POST/PRE ratios in the variability of C7 marker positions in both the A-P and M-L directions (*p* = 0.073 and *p* = 0.079, respectively), as well as in the variability of A-P C7 marker velocities (*p* = 0.085). These trends suggest more drift on the treadmill and increased stride-to-stride variability in A-P trunk movement following whole-body fatigue compared to localized fatigue. However, the POST/PRE ratio of mean peak COM velocity in the M-L direction (*p* < 0.001) was significantly greater after localized fatigue, indicating an increased magnitude of trunk sways.

## 4. Discussion

Our findings do not support our hypothesis that whole-body motor fatigue would have a more pronounced impact on gait variability and stability compared to localized motor fatigue. Contrary to our expectations, we observed significantly greater increases in mean step width, mean MOS_ML_, knee joint angle variability, and mean peak M-L COM velocity after localized fatigue than after whole-body fatigue. Notably, increasing step width or MOS_ML_ has been shown to be a common strategy to maintain balance during walking [31], suggesting that localized fatigue might have a greater effect on balance than whole-body fatigue. In addition, our results show that participants exhibited an improved local stability of trunk motion in both the A-P and ML directions following whole-body fatigue, whereas a similar level of local stability was maintained after localized fatigue, as observed in our previous study [12]. These results are consistent with those of Suzuki et al (2014) [16] and Lehnen et al (2020) [11], respectively, who found improved local stability following whole-body motor fatigue and similar local stability following localized fatigue when participants were required to maintain the same walking speed before and after fatigue. Our findings suggest that localized fatigue has a greater impact on gait variability and stability than whole-body fatigue.

While whole-body fatigue generally demonstrates less pronounced effects on gait compared to localized fatigue, our findings indicate a tendency, though not statistically significant, for whole-body fatigue to induce greater drifts in both the A-P and M-L directions. Additionally, we observed increased stride-to-stride upper body movement variability in the A-P direction in comparison to localized fatigue. Previous studies, although primarily focusing on standing postural stability rather than walking stability, have shown that visual contribution to standing postural stability deteriorates following whole-body exercises such as prolonged treadmill walking or running [22,23,35,36]. These exercises lead to increased postural sway [35,36,37], with the effect being more transient in the sagittal plane than in the frontal plane [35]. This observation might partially explain the increased drifts and A-P trunk movement variability following whole-body fatigue compared to localized fatigue. In addition to visual disturbance, repetitive head movements during prolonged walking may further disrupt the integration of vestibular information for balance control [22,23], contributing to the observed increases in drifts and trunk movement variability to some extent.

The observed improvement in the local stability of trunk motion following whole-body fatigue may be attributed to compensatory neuromuscular strategies activated in response to its widespread physiological demands (e.g., increased metabolic and cardiovascular load) associated with whole-body fatigue, in addition to muscle fatigue. These demands likely elicit systemic adaptations, such as enhanced coordination and sensory integration, that prioritize postural control and stability. Supporting this explanation, Suzuki et al. (2014) [16] found that prolonged walking, which induces whole-body fatigue, leads to the increased co-activation of the triceps surae and enhanced muscle synergies, potentially contributing to improved gait stability. In contrast, localized fatigue impacts specific muscle groups, potentially limiting the range of compensatory strategies available. Another possible explanation for the observed difference in walking stability and variability between the two fatigue conditions is the walking adaptation period. Gait adaptation to fatigue may have developed progressively during the whole-body fatiguing activity, allowing participants to make neuromuscular adjustments throughout the protocol. In contrast, for the localized fatigue condition, participants had to make these adjustments during the post-fatigued walking trials, without the opportunity for gradual adaptation during the fatigue process itself. Moreover, increased gait variability does not necessarily imply a loss of dynamic stability. It was hypothesized that motor variability in human movement consists of two components: ’bad’ variability, which arises from neuromuscular errors that can impair task performance or cause instability, and ’good’ variability, which reflects the central nervous system’s flexibility in adapting to unpredictable situations without compromising task performance or stability [38]. Similar findings have been reported previously where stability was maintained or even improved at the cost of increased variability [18,39,40]. This suggests that participants may adjust their walking patterns in response to fatigue, thereby increasing gait variability, but these adjustments may help preserve or enhance stability.

A reduction in force generation capacity, as indicated by maximum voluntary contraction (MVC), is commonly used to objectively quantify the degree of motor fatigue in protocols inducing localized fatigue. In contrast, previous protocols designed to induce whole-body fatigue often lacked the objective quantification of motor fatigue. These protocols typically relied solely on the subjective ratings of perceived exertion and/or voluntary exhaustion to terminate the exercise [41,42] or simply set an exercise time or distance [16,43,44]. In our study, comparing the effects of localized versus whole-body motor fatigue on gait, we aimed to reach a fatigue level corresponding to approximately a 20% reduction in maximum leg press force production in our prior investigation or a similar reduction in vertical jump height in the present study. It is possible that our findings, which suggest a greater impact of localized fatigue on gait variability and stability compared to whole-body fatigue, may not be directly applicable under highly fatigued conditions. While previous studies examining the effects of localized lower-extremity fatigue employed a wide range of fatigue levels [14,45], ranging from ~12% to 50% of reduction in MVC, our results are consistent with their overall findings regarding the gait parameters affected by motor fatigue. However, it is important to note that a direct comparison of gait changes resulting from different fatigue levels reported in previous studies may not be feasible due to variations in outcome measures and testing protocols (e.g., self-paced versus constant walking speed).

One major limitation of this study is that, unlike the whole-body fatigue study where the post-fatigued walking trials were conducted immediately following the fatiguing session, our previous localized fatigue study included additional post-fatigue walking trials to assess dual-task walking performance. Consequently, the walking-only condition was not executed right after the localized fatiguing exercise, potentially attenuating the observed localized fatiguing effects. However, despite the possibility of underestimating the effects of localized fatigue, we still observed a greater impact from localized fatigue than whole-body fatigue. Moreover, the walking trial lengths differed between the two studies. In the localized fatigue study, each trial was limited to 3 min to reduce the overall testing duration, as multiple post-fatigue walking trials were conducted. In the whole-body fatigue study, only one post-fatigue walking trial was conducted, allowing a duration of 5 min. However, since we compared POST/PRE ratios between the two studies/fatiguing protocols rather than directly comparing the raw values of the outcome measures, we believe that the influence from the differing trial lengths on the results is minimal. Another limitation of the study is the difference in methods used to validate fatigue intensity, specifically maximum leg press force production versus maximum vertical jump height. While we made efforts to standardize fatigue intensity across the two studies, this methodological difference may have influenced the observed outcomes, in addition to the effects of the different types of fatigue.

## 5. Conclusions

The current study investigated the effects of whole-body motor fatigue induced by incline treadmill walking on gait stability and variability and compared these effects with those of localized motor fatigue studied in a prior study. Our results demonstrate that localized fatigue has a greater impact on gait variability and stability than whole-body fatigue, showing greater alterations in mean step width, the mean mediolateral margins of stability, knee joint angle variability, and mean peak mediolateral COM velocity. Regarding walking stability, local stability was improved following whole-body fatigue, while a similar level was maintained following localized fatigue. Our study indicates that the nature of motor fatigue, whole-body versus localized, could lead to varying degrees of changes in gait performance.

## Figures and Tables

**Table 1:** Descriptive statistics for walking stability and variability parameters when walking at 1.25 m/s before (PRE) and after (POST) the whole-body motor fatiguing protocol induced by incline treadmill walking (mean ± STD). *p*-Values less than 0.05 are in bold.

Parameters	PRE	POST	*p*-Value	Cohen’s d
A-P local stability	0.199 ± 0.024	0.181 ± 0.025	**0.001**	0.79
M-L local stability	0.143 ± 0.040	0.128 ± 0.043	**0.039**	0.42
Mean MOS_AP_ (cm)	11.1 ± 2.7	11.9 ± 2.6	**<0.001**	0.92
STD MOS_AP_ (cm)	1.33 ± 0.38	1.37 ± 0.34	0.155	0.23
Mean MOS_ML_ (cm)	14.4 ± 2.1	14.4 ± 2.1	0.448	0.03
STD MOS_ML_ (cm)	1.11 ± 0.35	1.20 ± 0.37	**0.016**	0.52
Mean step length (cm)	62.8 ± 3.2	63.2 ± 3.0	0.058	0.37
STD step length (cm)	1.57 ± 0.43	1.47 ± 045	0.070	0.35
Mean step width (cm)	17.4 ± 3.8	16.5 ± 3.9	0.051	0.38
STD step width (cm)	2.29 ± 0.72	2.60 ± 0.90	**0.005**	0.64
Mean stride time (s)	1.108 ± 0.055	1.111 ± 0.052	0.284	0.13
STD stride time (s)	0.020 ± 0.007	0.018 ± 0.005	0.064	0.36
meanSD Ankle (°)	1.34 ± 0.27	1.31 ± 0.30	0.312	0.11
meanSD Knee (°)	1.82 ± 0.39	1.80 ± 0.35	0.344	0.09
meanSD Hip (°)	1.17 ± 0.27	1.20 ± 0.25	0.153	0.24
meanSD A-P COM position (cm)	6.01 ± 2.27	6.88 ± 1.95	**0.043**	0.40
meanSD M-L COM position (cm)	2.53 ± 0.71	3.33 ± 1.04	**<0.001**	0.92
meanSD A-P COM velocity (cm/s)	3.09 ± 0.78	3.14 ± 0.77	0.295	0.12
meanSD M-L COM velocity (cm/s)	2.71 ± 0.61	3.07 ± 0.77	**<0.001**	0.83
Mean peak A-P COM velocity (cm/s)	15.73 ± 3.50	16.45 ± 3.45	**0.006**	0.62
Mean peak M-L COM velocity (cm/s)	14.29 ± 2.09	13.71 ± 2.00	0.134	0.26

**Table 2:** Descriptive statistics for POST/PRE ratios of walking stability and variability parameters for the whole-body and localized fatigue protocols (mean ± STD). *p*-Values less than 0.05 are in bold.

POST/PRE Ratio	Whole-Body Fatigue	Localized Fatigue	*p*-Value	Cohen’s d
A-P local stability	0.91 ± 0.11	0.98 ± 0.07	**0.021**	0.69
M-L local stability	0.92 ± 0.23	0.98 ± 0.11	0.140	0.36
Mean MOS_AP_	1.08 ± 0.10	1.10 ± 0.17	0.449	0.16
STD MOS_AP_	1.04 ± 0.14	1.05 ± 0.12	0.423	0.06
Mean MOS_ML_	1.00 ± 0.05	1.03 ± 0.04	**0.015**	0.74
STD MOS_ML_	1.09 ± 0.17	1.04 ± 0.11	0.267	0.30
Mean step length	1.01 ± 0.02	1.01 ± 0.02	0.415	0.07
STD step length	0.95 ± 0.18	1.02 ± 0.13	0.094	0.44
Mean step width	0.96 ± 0.11	1.05 ± 0.08	**0.002**	0.98
STD step width	1.14 ± 0.22	1.08 ± 0.13	0.149	0.34
Mean stride time	1.00 ± 0.02	1.00 ± 0.02	0.256	0.22
STD stride time	0.94 ± 0.22	1.02 ± 0.10	0.078	0.47
meanSD Ankle	0.99 ± 0.14	1.02 ± 0.12	0.202	0.28
meanSD Knee	1.00 ± 0.11	0.11 ± 0.09	**<0.001**	1.13
meanSD Hip	1.04 ± 0.13	1.10 ± 0.13	0.064	0.51
meanSD A-P C7 position	1.23 ± 0.38	1.03 ± 0.44	0.073	0.49
meanSD M-L C7 position	1.33 ± 0.33	1.18 ± 0.30	0.079	0.47
meanSD A-P C7 velocity	1.15 ± 0.18	1.09 ± 0.11	0.085	0.46
meanSD M-L C7 velocity	1.13 ± 0.21	1.13 ± 0.13	0.459	0.03
Mean peak A-P COM velocity	1.05 ± 0.08	1.04 ± 0.08	0.373	0.11
Mean peak M-L COM velocity	0.97 ± 0.14	1.13 ± 0.13	**<0.001**	1.18

## Data Availability

Data are contained within the article.
